# GLI2 promotes cell proliferation and migration through transcriptional activation of *ARHGEF16* in human glioma cells

**DOI:** 10.1186/s13046-018-0917-x

**Published:** 2018-10-11

**Authors:** Dengliang Huang, Yiting Wang, Linlin Xu, Limin Chen, Minzhang Cheng, Wei Shi, Huanting Xiong, Detina Zalli, Shiwen Luo

**Affiliations:** 1 Center for Experimental Medicine, The First Affiliated Hospital of Nanchang University, 17 Yongwai Street, Nanchang, 330006 Jiangxi China; 2 Jiangxi Key Laboratory of Molecular Diagnostics and Precision Medicine, Nanchang, 330006 Jiangxi China; 3 School of Biological and Chemical Sciences, Queen Mary University of London, Mile End Road, London, E1 4NS UK

**Keywords:** Hedgehog signaling, GLI, ARHGEF16, Glioma, Migration, Proliferation

## Abstract

**Background:**

The Hedgehog (Hh) signaling pathway plays critical roles in modulating embryogenesis and maintaining tissue homeostasis, with glioma-associated oncogene (GLI) transcription factors being the main mediators. Aberrant activation of this pathway is associated with various human malignancies including glioblastoma, although the mechanistic details are not well understood.

**Methods:**

We performed a microarray analysis of genes that are differentially expressed in glioblastoma U87 cells overexpressing GLI2A, the active form of GLI2, relative to the control cells. Chromatin immunoprecipitation and dual-luciferase assays were used to determine whether Rho guanine nucleotide exchange factor 16 (ARHGEF16) is a downstream target of GLI2. Then, transwell migration, EdU and soft-agar colony formation assays were employed to test effects of ARHGEF16 on glioma cancer cell migration and proliferation, and the effects of GLI2/ARHGEF16 signaling on tumor growth were examined in vivo. Finally, we performed yeast two-hybrid assay, Co-IP and GST-pull down to identify factors that mediate effects of ARHGEF16.

**Results:**

We found that ARHGEF16 mRNA level was upregulated in U87 cells overexpressing GLI2A relative to control cells. GLI2 binds to the *ARHGEF16* promoter and activates gene transcription. Glioma cells U87 and U118 overexpressing ARHGEF16 showed enhanced migration and proliferation relative to the control cells, while knockdown of ARHGEF16 in H4 cells led to decreased cell proliferation compared to the control H4 cells. In contrast to the promoting effect of GLI2A overexpression on glioma xenograft growth, both GLI2 inhibition and ARHGEF16 knockdown retarded tumor growth. Cytoskeleton-associated protein 5 (CKAP5) was identified as an interaction protein of ARHGEF16, which is important for the stimulatory effects of ARHGEF16 on glioma cell migration and proliferation.

**Conclusions:**

These results suggest that therapeutic strategies targeting the GLI2/ARHGEF16/CKAP5 signaling axis could inhibit glioma progression and recurrence.

**Electronic supplementary material:**

The online version of this article (10.1186/s13046-018-0917-x) contains supplementary material, which is available to authorized users.

## Background

The Hedgehog (Hh) signaling pathway is an important regulator of embryonic development and homeostasis in metazoans [1, 2]. In vertebrates, the main components include Hh ligand, the membrane receptor Patched (PTCH), signal transducer protein Smoothened (SMO), negative regulator suppressor of fused (SuFu), and glioma-associated oncogene (GLI) transcription factors including GLI1, GLI2, and GLI3 [3]. In germ cells, dysregulation of Hh leads to various congenital abnormalities such as Greig cephalopolysyndactyly syndrome and Pallister-Hall syndrome caused by deleterious mutations in *Gli3* [4, 5], as well as holoprosencephaly-like features and pituitary anomalies resulting from loss-of-function mutations in *GLI2* [6]. Additionally, aberrant activation of Hh signaling in somatic cells has been implicated in human cancers [7] including basal cell carcinoma [8], medulloblastoma [9], lung cancer [10], breast cancer [11], and glioma [12]. Excess Hh ligand expressed by cancer or stromal cells, inactivating mutations in PTCH or SuFu, and activating mutations in SMO can all lead to derepression of GLI [13] and inappropriate activation of target gene transcription [14, 15]. These genes regulate cellular processes associated with tumorigenesis, including tumor cell survival/proliferation and metastasis and cancer stem cell self-renewal [14, 15]. As such, various inhibitors of Hh signaling components have been developed for cancer therapy [16–18].

Glioma arises from neurogliocytes and is a common type of central nervous system neoplasm. Around 54% of glioma cases are classified as glioblastoma (World Health Organization grade IV glioma) [19, 20], which is difficult to treat; even with early diagnosis and aggressive surgery and radio−/chemotherapy, the median survival of these patients is 15 months [21], with a 5-year survival of just 5% [22, 23]. This is due to the malignant behaviors of glioma stem cells—including proliferation, angiogenesis, and invasiveness—that are modulated by Hh signaling [12, 24]. Combined inhibition of Hh and Notch pathways sensitizes cluster of differentiation (CD) 133^+^ glioma stem cells to chemotherapy [25], while targeted inhibition of the Hh pathway improved the survival of glioma xenograft model mice [26].

Rho GTPases modulate cell morphogenesis, proliferation, invasion, and survival through regulation of the actin cytoskeleton [27, 28]. Most Rho GTPases identified to date (e.g., RhoA, RhoC, Rac1, and Cdc42) have oncogenic functions when abnormally activated. For example, loss of RhoC inhibited cancer cell metastasis in a RhoC^−/−^; pyV-MT mouse model of mammary tumors [29], and knocking out one allele of the *Rac1* gene impaired K-Ras-induced oral papilloma growth [30]. The switch between GDP-bound inactive and GTP-bound active states of Rho proteins is mediated by GTPase-activating proteins (GAP) and guanine nucleotide exchange factors (GEFs) [31]. GAPs accelerate GTP hydrolysis by Rho proteins; formation of GDP-bound Rho proteins block Rho GTPase signaling. On the other hand, GEFs facilitate the conversion of GDP-bound inactive Rho proteins to a GTP-bound active form by overriding the inhibitory effects of GDP dissociation inhibitors; thus, GEFs are generally considered to be pro-oncogenic. ARHGEF16 (also known as Ephexin4, GEF16, or NBR) is a GEF that can activate RhoG, Rac1, and Cdc42 proteins of the Rho GTPase family [32–34] and thereby promote migration and resistance to apoptosis of breast cancer cells [35] independent of Ephrin signaling. However, the mechanism underlying the functions of ARHGEF16 is not fully understood.

In this study, we identified ARHGEF16 as a target gene of GLI2 that interacts with cytoskeleton-associated protein 5 (CKAP5) to regulate glioma cell migration and proliferation, thus promoting glioma progression.

## Methods

### Reagents, antibodies, and constructs

The GLI inhibitor GANT61 and protease inhibitor cocktail were purchased from Sigma-Aldrich (St. Louis, MO, USA). Puromycin was from Genechem (Shanghai, China) and Solarbio (Beijing, China), respectively. Lipofectamine 2000 transfection reagent (#11668019) and TRIzol reagent (#15596018) were from Thermo Fisher Scientific (Waltham, MA, USA). Protein A agarose beads (#11134515001) and Protein G agarose beads (#11243233001) were from Roche (Palo Alto, CA, USA), and Glutathione Sepharose 4B beads (#17–0756-01) were from GE Healthcare (Little Chalfont, UK). Antibodies against the following proteins were used for western blotting: ARHGEF16 (ab86068), GLI1 (ab49314), GLI2 (ab26056), SMO (ab38686), SuFu (ab52913), PTCH1 (ab55629), CKAP5 (ab86073), and normal rabbit IgG (ab171870) (all from Abcam, Cambridge, MA, USA); Forkhead box M1 (Abgent, San Diego, CA, USA; AT2097a); glyceraldehyde 3-phosphate dehydrogenase (Millipore, Billerica, MA, USA; MAB374); β-actin (Santa Cruz Biotechnology, Santa Cruz, CA, USA; sc-1616-R); and Flag (F3165) and c-Myc (M4439) (Sigma-Aldrich). Antibodies against GLI2 (sc-271786) used in the chromatin immunoprecipitation (ChIP) assay was purchased from Santa Cruz Biotechnology. All other chemicals used were of analytical grade and were purchased from Sigma-Aldrich. A reverse transcription kit (Takara Bio, Otsu, Japan; RR047A) and real-time quantitative (q)PCR assay kit (Takara Bio; RR820A) were used for mRNA quantitation. Cell-light™ EdU Apollo567 In Vitro Kit (Cat #: C10310–1) was purchased from Guangzhou RiboBio Co., LTD in China.

Luciferase reporter constructs used to examine transcriptional activation of ARHGEF16 by GLI2 with the dual-luciferase assay were generated by inserting *ARHGEF16* promoter sequences into the pGL3-basic vector. Primers used to generate the three reporter constructs are shown in Additional file 1: Table S1. pCMV6-Entry-Gli2-MYC/DDK (RC217291) containing human *GLI2* mRNA (NM_005270) was purchased from OriGene (Rockville, MD, USA). The first 984 bases of *GLI2* mRNA were deleted with a mutagenesis kit (Toyobo, Osaka, Japan; SMK-101) to generate a constitutively active form of GLI2 (GLI2A) lacking amino acids 1–328 [36]. Human *ARHGEF16* mRNA (NM_014448) was inserted into pGBKT7 and pGEX-6p-1 plasmids using the In-Fusion Cloning kit (Clontech Laboratories, Mountain View, CA, USA; #639619) to generate pGBKT7-ARHGEF16 and pGEX-6p-1-ARHGEF16, respectively. *ARHGEF16* or *CKAP5* silencing constructs were generated with the BLOCK-iT Pol II miR RNAi Expression Vector kit (Invitrogen, Carlsbad, CA, USA; K4936–00); lentiviruses (LVs) expressing GLI2A or ARHGEF16 or for *ARHGEF16* knockdown were obtained from GeneChem (Shanghai, China). GV358 and GV307 LV vectors were used for overexpression or knockdown, respectively; the target sequences are shown in Additional file 1: Table S2. The target sequences for *GLI2* knockdown was TCCTGAACATGATGACCTA [37].

### Cell culture and transfection

H4, U87, and U118 human glioma cell lines and 293 T human embryonic kidney cell line were purchased from the American Type Culture Collection (Manassas, VA, USA). Cells were cultured in Dulbecco’s Modified Eagle’s Medium (DMEM; Gibco, Grand Island, NY, USA) supplemented with 10% fetal bovine serum (FBS; Gibco), 100 U/ml penicillin, and 100 μg/ml streptomycin at 37 °C in a humidified incubator of 5% CO_2_. Cells were transiently transfected with polyetherimide for 293 T cells to detect the efficiency of sh-ARHGEF16 or sh-CKAP5 constructs (Additional file 2: Figure S1A-C), or with Lipofectamine 2000 for glioma cell lines according to the manufacturer’s instructions. LV systems were used to establish stable glioma cell lines overexpressing GLI2A or ARHGEF16 or to knock down *GLI2* or *ARHGEF16*. Puromycin (0.5 μg/ml) was added to cultures to maintain stable overexpression in the cell lines.

### Microarray analysis

Microarray analysis was performed by Compass Biotechnology (Beijing, China). Briefly, total RNA was extracted from U87 cells stably overexpressing GLI2A (U87 GLI2A) and control cells (U87 Control) using TRIzol reagent, and then processed for hybridization to an HT-12 v4 Expression BeadChip (Illumina, San Diego, CA, USA). The array was washed and then scanned using an Illumina BeadArray Reader. Differentially expressed genes (DEGs) between LV-Control and LV-GLI2A U87 cells were identified from the data.

### Western blotting and real-time qPCR

Total protein was extracted from cultured cells using lysis buffer (0.5% Lubrol-PX, 50 mM KCl, 2 mM CaCl_2_, 20% glycerol, 50 mM Tris-HCl [pH 7.4], and 1% protease inhibitor cocktail), and the relative levels of target proteins were evaluated by immunoblotting. For qPCR, total RNA was extracted from cultured cells using TRIzol reagent and 1 μg of total RNA was used for reverse transcription and real-time qPCR on an ABI StepOne Plus detection system (Applied Biosystems, Foster City, CA, USA). Sequences of primers for detecting each target gene are shown in Additional file 1: Table S3.

### Dual luciferase assay

U87 cells grown to 70% confluence in 24-well plates were transfected in triplicate with 0.75 μg of pGL3-basic-ARHGEF16 promoter-luciferase reporter and 0.25 μg of GLI expression plasmid or empty vector along with 0.025 μg of pRL-TK for normalization. After 48 h, luciferase activity was measured with a luminometer using the dual-luciferase assay kit (Promega, Madison, WI, USA; TM040) according to the manufacturer’s instructions. The activity of pGL3-basic-ARHGEF16 promoter-luciferase reporter normalized to that of pRL-TK Rluc reporter was compared between U87 cells transfected with GLI expression plasmid or empty vector.

### ChIP assay

H4 cells were cross-linked with 1% (*v*/v) formaldehyde in phosphate-buffered saline for 10 min at 37 °C with gentle shaking. After adding 0.125 M glycine to terminate the reaction, the cells were lysed with lysis buffer on ice. Chromatin DNA was sheared by sonication to obtain ~ 500 bp fragments that were then mixed with anti-GLI2 antibody and protein G-agarose to enrich DNA fragments bound to GLI2 through immunoprecipitation. After decrosslinking, the precipitated DNA was analyzed by qPCR to assess the *ARHGEF16* promoter regions containing putative GLI binding sites.

### Cell migration and proliferation assays

The cell migration assay was performed using transwell plates (8 μm pore size, 6.5 mm diameter; Corning Life Sciences, Lowell, MA, USA). Briefly, 2 × 10^4^ cells in 200 μl of 2% FBS DMEM were seeded into the upper chamber of the transwell insert, while the bottom chamber was filled with 800 μl of 10% FBS DMEM. After 24 h, cells on the upper surface of the membrane were removed and those on the lower surface of the membrane were fixed with 4% paraformaldehyde and stained with Crystal Violet. Cells were observed using an optical microscope after washing to remove excess dye and quantified using ImageJ software (National Institutes of Health, Bethesda, MD, USA). The cells over the whole filter were counted while one field of each filter was presented in the figures.

Proliferative capacity of U87 and U118 glioma cells was examined with the soft agar colony formation assay. A 400-μl volume of 0.5% agar solution containing 10% FBS DMEM was added each well of a 12-well plate, and 200 μl of 0.6% agar solution was mixed with 200 μl of 20% FBS DMEM containing 2 × 10^3^ cells and added to the top of the solidified 0.5% agar. A 200-μl volume of 10% FBS DMEM was added to the wells, which was replenished every three days, and the cells were incubated for 2 weeks; colonies with a diameter larger than 50 μm were counted.

H4 cell proliferation was assessed with plate colony formation, where 1.5 × 10^3^ H4 sh-Control or H4 sh-ARHGEF16 cells were seeded in one well of the 6-well plate and cultured with 10% FBS DMEM for about 2 weeks. Then cell colonies, fixed with 4% paraformaldehyde and stained with Crystal Violet, were examined and photographed under a phase-contrast microscope and quantified using ImageJ software. Furthermore, Cell-light™ EdU Apollo567 In Vitro Kit was used for cell proliferation analysis following the manufacturer’s instructions.

### Subcutaneous xenograft assay

Female BALB/c nu/nu athymic nude mice (4 weeks old) were used for experiments according to Guidelines for the Welfare of Animals in Experimental Neoplasia published by The United Kingdom Coordinating Committee on Cancer Research. The experimental protocol was approved by the Institutional Animal Care and Use Committee of Nanchang University and regional authorities. A total of 5 × 10^6^ Control+sh-Control, GLI2A + sh-Control or GLI2A + sh-ARHGEF16 U87 cells were injected into the flanks of each of five nude mice to examine the role of GLI2/ARHGEF16 signaling on glioma growth in vivo, and tumor formation was observed starting 6 days later. The size of glioma xenografts was measured every two to three days, and tumor volumes were calculated by the formula: 0.5 × length×width×(length + width) [38]. Twenty-four days after injecting the U87 cells, mice were sacrificed under anesthesia and the tumor xenografts were harvested for immunohistochemical analysis.

Furthermore, to address the GLI2 inhibition effects in a pre-clinical context, GLI2A or Control U87 cells were inoculated into ten nude mice as above, and then the mice loaded with GLI2A and Control U87 xenografts were randomly divided into two groups and treated with only solvent (corn oil: ethanol, 4:1) or GANT61 (25 mg/kg) in solvent through intraperitoneal injection every second day for 4 weeks [18, 39]. In addition, 5 × 10^6^ sh-Control or sh-ARHGEF16 U118 stable cell lines were inoculated in five nude mice as above to examine the effects of ARHGEF16 knockdown on tumor growth.

### Protein-protein interaction assay

Cells were solubilized in lysis buffer containing 0.5% Lubrol-PX, 50 mM KCl, 2 mM CaCl_2_, 20% glycerol, 50 mM Tris-HCl (pH 7.4), and protease inhibitors. Pre-cleared cell lysate containing Flag-ARHGEF16 was incubated with 30 μl of 1:1 slurry magnetic beads conjugated with anti-Flag antibody for 4 h at 4 °C. The beads were washed four times with lysis buffer before adding sodium dodecyl sulfate (SDS) sample buffer, and the samples were analyzed by western blotting. To detect the interaction between ARHGEF16 and CKAP5, glutathione S- transferase (GST)-ARHGEF16 fusion protein was expressed in BL21 bacterial cells, purified, and immobilized on Glutathione Sepharose 4B beads (Amersham Pharmacia, Piscataway, NJ, USA), and then incubated with lysate from U87 cells. Bead-associated proteins were subjected to SDS-polyacrylamide gel electrophoresis and immunoblotting. Experiments were repeated at least three times.

### Statistical analysis

Quantitative data are presented as mean ± SD of at least three experiments. Differences between groups were assessed with the Student’s *t*-test or by one-way analysis of variance and were considered statistically significant at *P* < 0.05 and highly significant at *P* < 0.01. Data were analyzed using SPSS v.17.0 software (SPSS Inc., Chicago, IL, USA).

## Results

### ARHGEF16 is positively regulated by GLI2

**Fig. 1:**
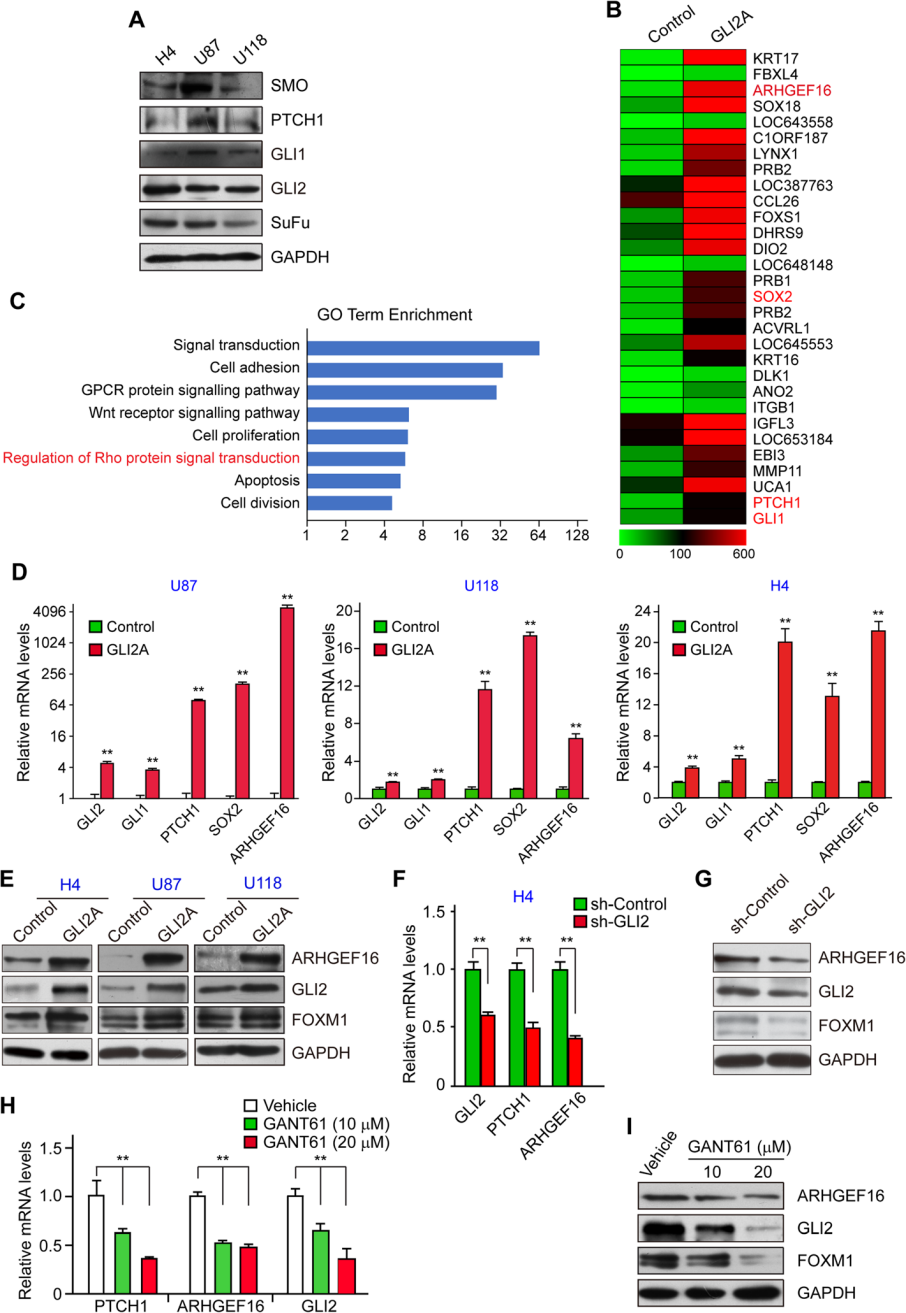
*ARHGEF16* is positively regulated by GLI2 in glioma cells. **a** Detection of Hh signaling pathway components PTCH1, SMO, SuFu, GLI1, and GLI2 in H4, U87, and U118 glioma cell lines. **b** Top upregulated genes in GLI2A U87 cells shown as a heat map, including the validated Hh signaling pathway target genes SOX2, GLI1, and PTCH1. **c** Gene Ontology term enrichment analysis of the major cellular activities associated with DEGs between Control and GLI2A U87 cells. **d**, **e** *ARHGEF16* mRNA (**d**) and protein (**e**) levels in GLI2A-overexpressing and control H4, U87, and U118 cells. *n* = 3, **, *P* < 0.01. **f**, **g** *ARHGEF16* mRNA (**f**) and protein (**g**) levels in *Gli2* knockdown and control H4 cells. *n* = 3, **, *P* < 0.01. **h**, **i** *ARHGEF16* mRNA (**h**) and protein (**i**) levels in H4 cells treated with indicated concentrations of the GLI2 inhibitor GANT61. n = 3, **, *P* < 0.01

The expression of the main Hh pathway components in H4, U87, and U118 glioma cell lines was confirmed by western blotting (Fig. 1a). To identify novel target genes of GLI transcription factors, we overexpressed GLI2A-a constitutively active form of GLI2-by LV infection in GLI2A U87 glioma cells and compared the gene expression profiles with that of U87 Control cells by microarray analysis. A total of 814 genes were downregulated and 1121 were upregulated in GLI2A U87 cells as compared to control cells; some of the most significantly upregulated genes are shown as heat map (Fig. 1b). Among these DEGs were the GLI target genes GLI1, PTCH1, and SOX2, which validated the microarray results. ARHGEF16 ranked third among genes upregulated in U87 GLI2A cells, and the Gene Ontology term enrichment analysis revealed “regulation of Rho protein signal transduction” as one of the major cellular activities associated with the DEGs (Fig. 1c). Based on these results, we selected ARHGEF16 as a candidate gene regulated by GLI2 affecting cell proliferation and migration in glioma.

We examined ARHGEF16 mRNA and protein levels in H4, U87, and U118 cells to confirm that it is a regulatory target of GLI2. When GLI2A was overexpressed in these cell lines, ARHGEF16 expression levels increased (Fig. 1d, e). Conversely, transcript and protein levels decreased upon *GLI2* knockdown in H4 cells (Fig. 1f, g). When GLI2 was inhibited in H4 cells by treatment with GANT61 (a small-molecule antagonist of GLI) [18], ARHGEF16 mRNA and protein levels were downregulated (Fig. 1h, i). These results indicate that GLI2 positively regulates ARHGEF16 expression in glioma cells.

### GLI2 directly activates ARHGEF16 transcription

**Fig. 2:**
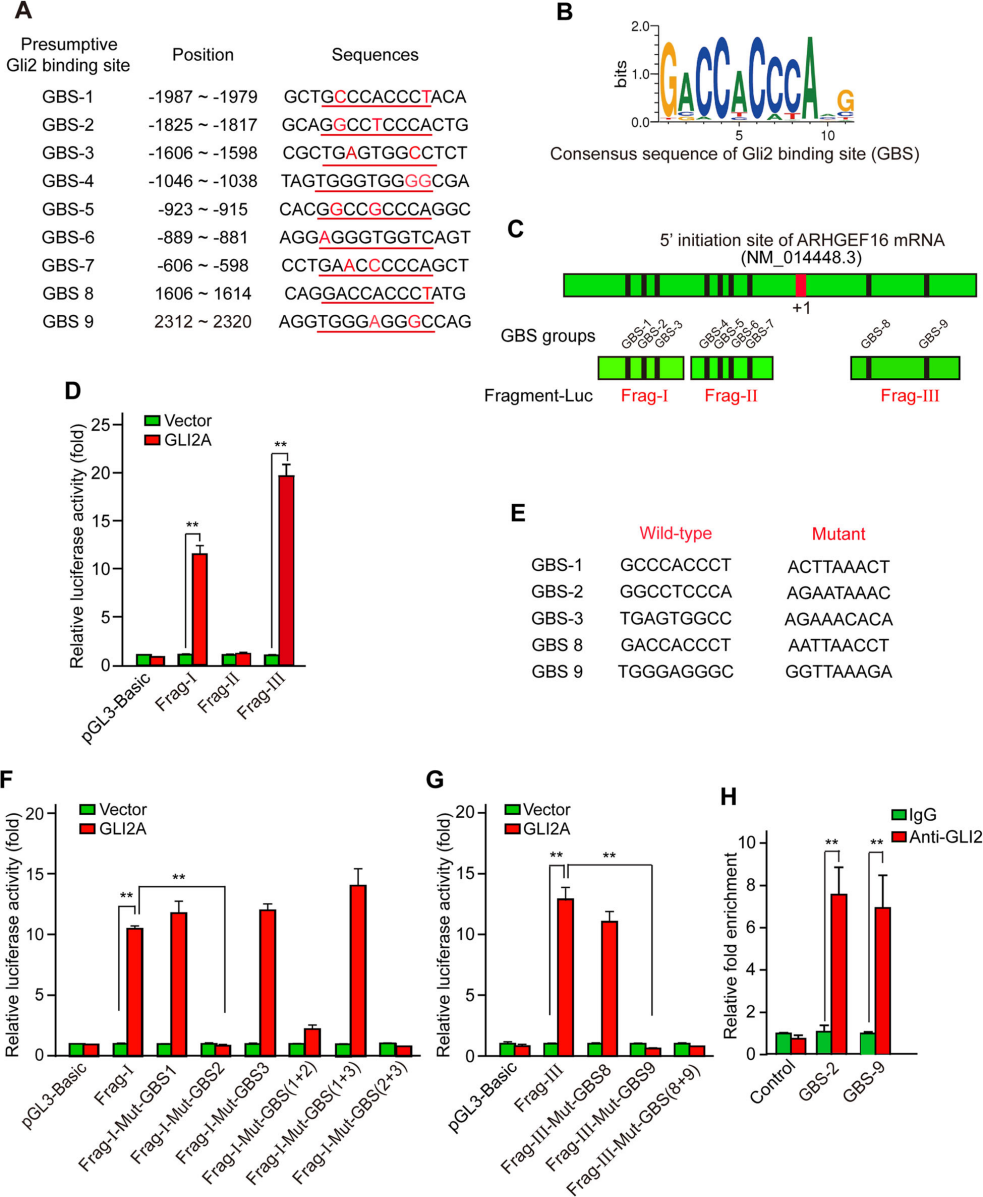
GLI2 directly activates *ARHGEF16* transcription. **a** Putative GBS within the genomic sequence adjacent to the TSS of the *ARHGEF16* gene. The GBS core sequence is underlined and bases other than the GBS consensus sequence are shown in red. **b** Consensus sequence of the GLI-binding site. **c** Schematic illustration of the distribution of GBS candidates within the *ARHGEF16* promoter and luciferase reporter constructs Frag-I, -II, and -III containing indicated GBS. **d** Dual-luciferase assay for detecting the activating effects of GLI2 on Frag-I, -II, and -III reporter constructs in GLI2A and the control U87 cells. n = 3, **, *P* < 0.01. **e** Mutant GBS sequences in Frag-I and -III reporter constructs. **f** Dual-luciferase assay for Frag-I and its variants in Control and GLI2A U87 cells. “M plus GBS number” denotes the mutated GBS in the luciferase reporter mutants. n = 3, **, *P* < 0.01. **g** Dual-luciferase assay for Frag-III and its variants in Control and GLI2A U87 cells. n = 3, **, *P* < 0.01. **h** ChIP assay in H4 glioma cells to assess the binding of GLI2 to the *ARHGEF16* promoter via GBS-2 and -9. n = 3, **, *P* < 0.01

We next used an online tool (www.genomatix.de) to identify putative GLI-binding sites (GBS) in the genomic sequence adjacent to the transcription start site (TSS) of the *ARHGEF16* gene to determine whether GLI2 binds to the *ARHGEF16* promoter and directly activates its transcription. We found nine putative GBS (Fig. 2a, b) within the − 2500 to + 2500 genomic region relative to the 5′ initiation site of *ARHGEF16* (NM_014448.3), which was numbered as + 1.

Genomic sequences containing the GBS were cloned into the pGL3 basic vector for the dual-luciferase assay, yielding the luciferase reporter constructs Frag-I, -II, and -III containing GBS 1–3, GBS 4–7, and GBS 8–9 (Fig. 2c). The constructs along with pRL-TK for normalization were co-transfected into U87 LV-GLI2A or LV-control cells, and luciferase activity was measured 48 h later. The Frag-I and -III constructs showed high luciferase activity in LV-GLI2A U87 cells (Fig. 2d). Since both constructs contained more than one GBS, we mutated each of these in turn and assayed luciferase activity (Fig. 2e). When GBS-2 in Frag-I (Fig. 2f) or GBS-9 in Frag-III (Fig. 2g) was mutated, induction of luciferase activity by GLI2 was completely abolished, indicating that these two sites are critical for activation of *ARHGEF16* transcription by GLI2. Accordingly, a ChIP assay in H4 glioma cells showed that compared to normal IgG, anti-GLI2 antibody enriched the chromatin fragments containing GBS-2 or − 9, while the negative control chromatin fragment could not be enriched by the antibody (Fig. 2h), implying that GLI2 specifically bound to the *ARHGEF16* promoter via both the GBS-2 and -9. Together, these results identify *ARHGEF16* as a novel direct target gene of GLI2 transcription factor.

### ARHGEF16 promotes glioma cell migration and proliferation

**Fig. 3:**
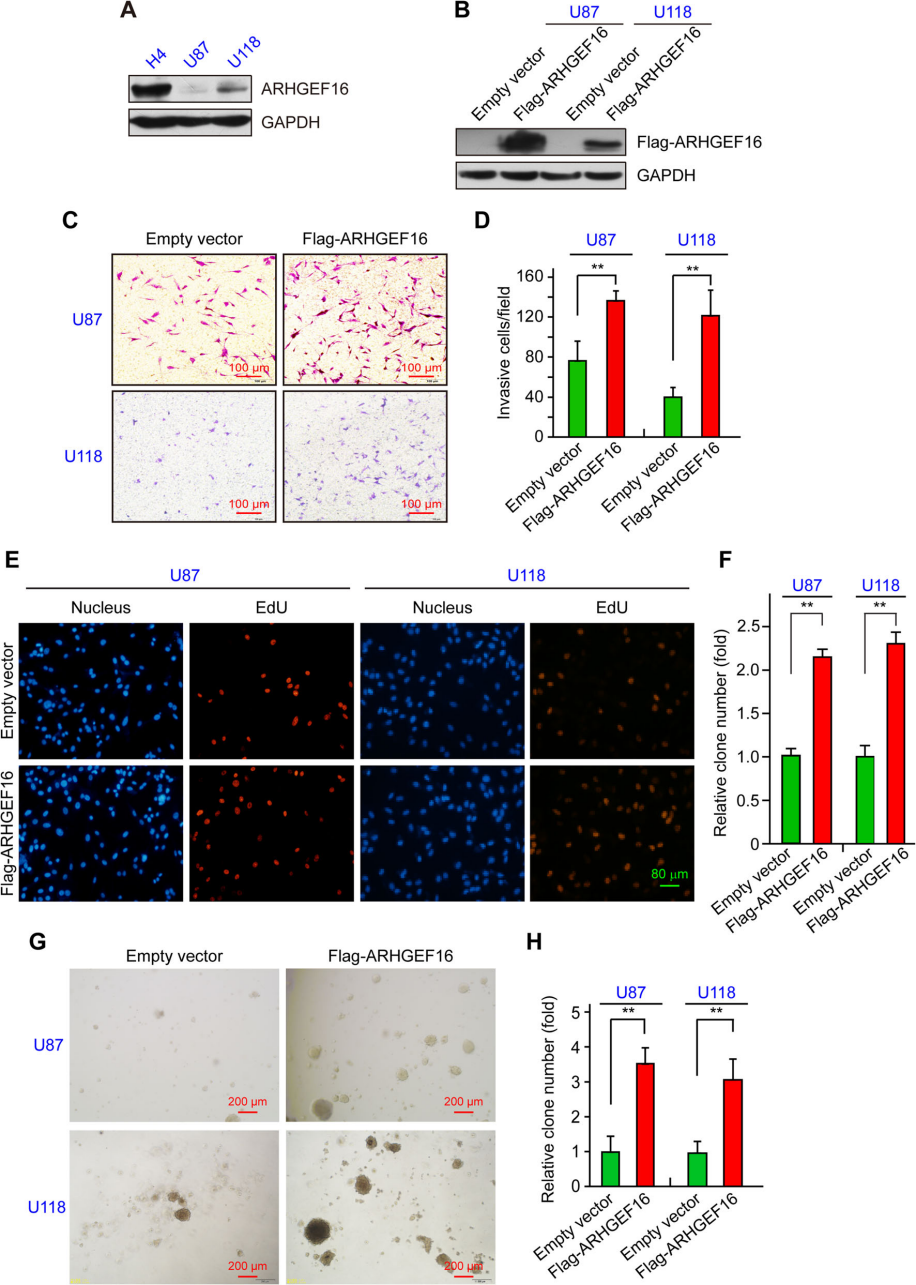
ARHGEF16 promotes migration and proliferation of glioma cells. **a** Detection of endogenous *ARHGEF16* protein level in H4, U87, and U118 cells by western blotting. **b** Validation of glioma cell lines stably overexpressing ARHGEF16. ARHGEF16 protein level was compared between Flag-ARHGEF16 U87 or Flag-ARHGEF16 U118 cells and corresponding control cells. **c**, **d** Comparison of the migratory potential between ARHGEF16 U87 or ARHGEF16 U118 cells and their corresponding control cells with the transwell migration assay. n = 3, **, *P* < 0.01. **e-h** Comparison of the proliferative capacity of ARHGEF16 U87 or ARHGEF16 U118 cells and their corresponding control cells with EdU assay (**e**, **f**) and soft agar colony formation assay (**g**, **h**). n = 3, **, *P* < 0.01

Our preliminary experiments showed that endogenous expression of ARHGEF16 was much higher in H4 cells than in U87 and U118 cells, with the latter two cell lines showing comparable levels (Fig. 3a). An LV system was used to overexpress the ARHGEF16 in both U87 and U118 cells. ARHGEF16 expression was increased in Flag-ARHGEF16 U87 and U118 cells relative to the corresponding control cells (Fig. 3b).

We carried out transwell migration, EdU and soft-agar colony formation assays to compare the migratory and proliferative potentials of ARHGEF16 U87 or U118 cells and their respective control cells. ARHGEF16 overexpression increased the number of U87 or U118 cells that migrated through the transwell insert membrane as compared to the corresponding control cells (Fig. 3c, d). In EdU assays, more cells in S phase of cell cycle were marked in ARHGEF16-overexpressing cells compared to the control cells (Fig. 3e, f), suggesting that more cells entered S phase from G1 phase for cell cycle progression when ARHGEF16 was overexpressed, and ARHGEF16-overexpressing cells formed more and larger colonies than the control groups in soft-agar colony formation assay (Fig. 3g, h). In combination, colony formation and EdU assays showed proliferation-promoting effects of ARHGEF16 on glioma cell proliferation. These results indicate that ARHGEF16 promotes glioma cell migration and proliferation.

### ARHGEF16 knockdown suppresses glioma growth

**Fig. 4:**
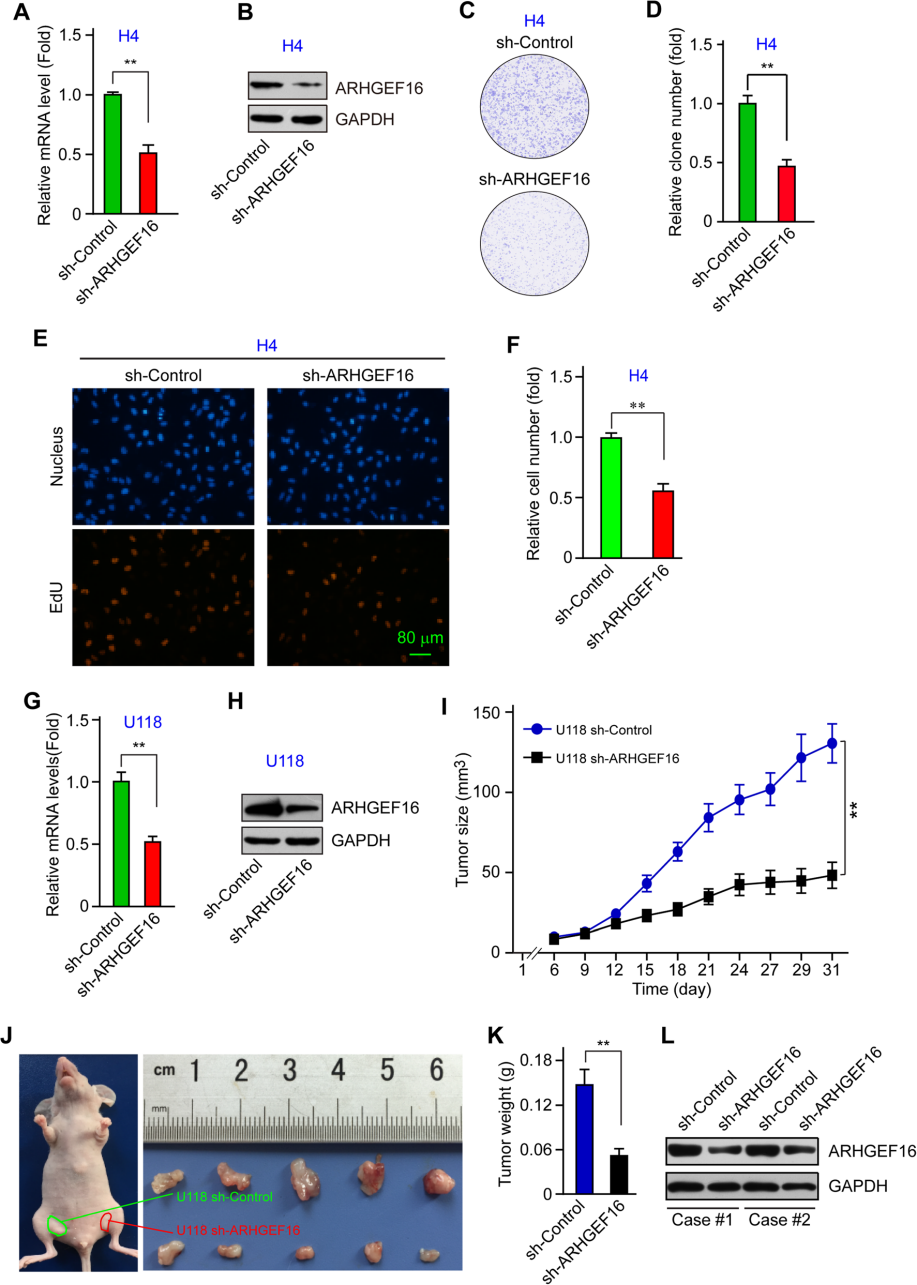
ARHGEF16 knockdown suppresses glioma growth. **a**, **b** Validation of ARHGEF16 knockdown in H4 cells; ARHGEF16 mRNA (**a**) and protein (**b**) levels were compared between sh-Control cells and sh-ARHGEF16 H4 cells. n = 3, **, *P* < 0.01. **c-f** Comparison of the proliferative capacity between sh-ARHGEF16 and control H4 cells with plate clone formation assay (**c**, **d**) and EdU assay (**e**, **f**). n = 3, **, *P* < 0.01. **g**, **h** ARHGEF16 mRNA (**g**) and protein (**h**) levels were detected to validate ARHGEF16 knockdown in U118 cells. n = 3, **, *P* < 0.01. **i** Growth curves of the U118 sh-Control and U118 sh-ARHGEF16 xenografts in nude mice. *n* = 5, **, *P* < 0.01. **j** Harvested glioma xenografts formed by the indicated U118 glioma cell lines. **k** Weight of the indicated glioma xenograft tumors. n = 5, **, *P* < 0.01. **l** The decreased ARHGEF16 protein level in U118 sh-ARHGEF16 xenografts compared to U118 sh-Control xenografts via western blotting

In contrast to the increased migration and proliferation capacities of ARHGEF16-overexpressing U87 or U118 cells, *ARHGEF16* knockdown in H4 cell (Fig. 4a, b) greatly inhibited glioma cell proliferationas assessed via plate colony formation (Fig. 4c, d) and EdU (Fig. 4e, f) assays. For in vivo study, given the weak oncogenic capacity of H4 cell and the low endogenous ARHGEF16 level in U87 cell, sh-Control and sh-ARHGEF16 U118 stable cell lines (Fig. 4g, h) were inoculated in athymic nude mice as described in methods section to determine ARHGEF16-knockdown effects on glioma progression. Tumor xenografts developed more slowly (Fig. 4i) in sh-ARHGEF16 group than in sh-Control group to generate smaller tumor xenografts (Fig. 4j, k), indicating retarded glioma progression by ARHGEF16 knockdown, and decreased ARHGEF16 protein level in sh-ARHGEF16 xenografts relative to that in the control group was confirmed via western blotting (Fig. 4l). These results indicate that ARHGEF16 knockdown suppressed glioma growth.

### GLI2/ARHGEF16 signaling promotes glioma progression

**Fig. 5:**
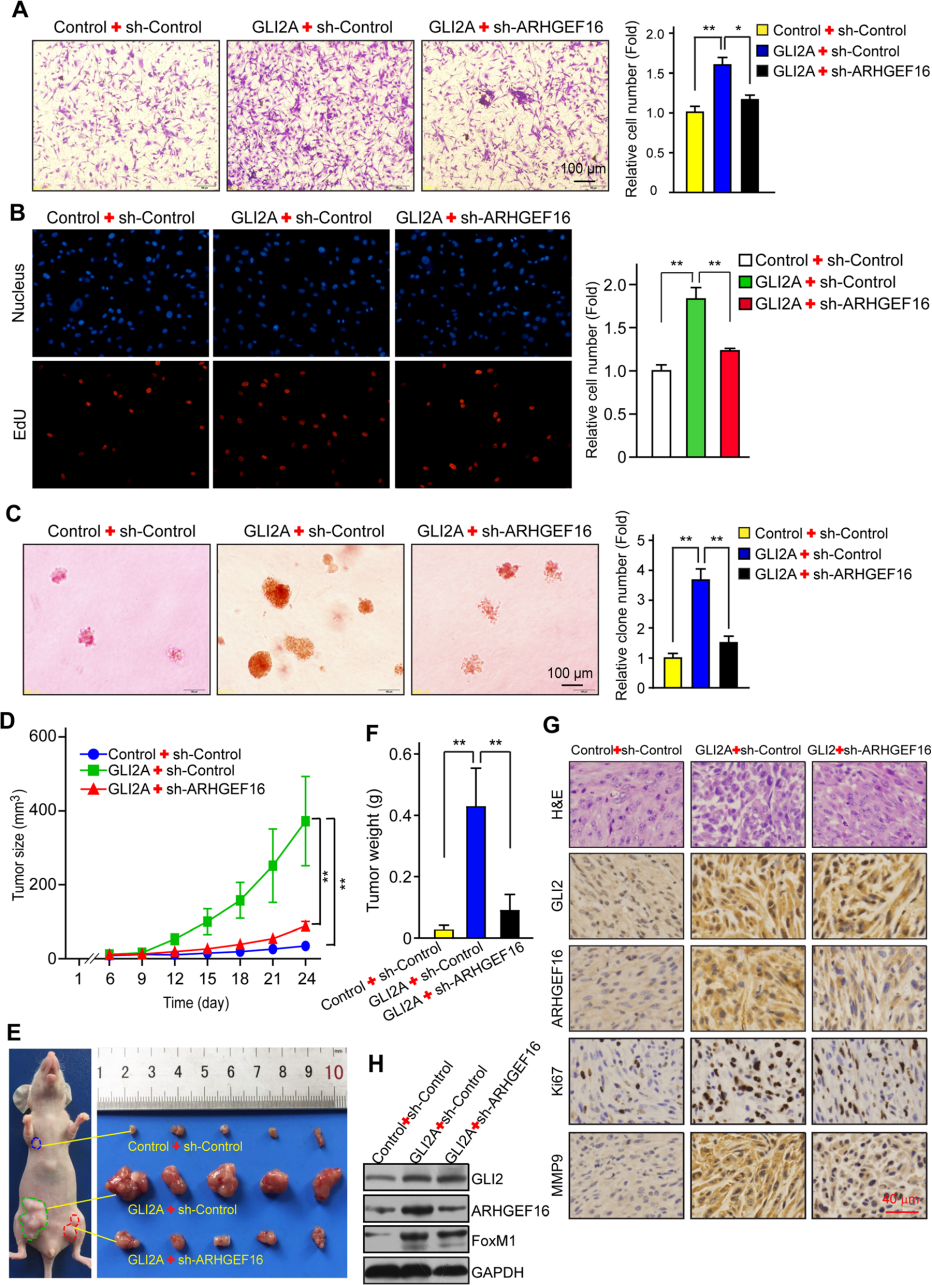
GLI2/ARHGEF16 signaling promotes glioma progression. **a-c** Migration (**a**) and proliferation (**b**, **c**) in Control+sh-Control, GLI2A + sh-Control and GLI2A + sh-ARHGEF16 U87 cells were compared with the transwell migration, EdU and soft-agar colony formation assays, respectively. n = 3, *, *P* < 0.05; **, *P* < 0.01. **d** Growth curves of xenografts formed by the indicated U87 cells in nude mice. n = 5, **, *P* < 0.01. **e** Image of the indicated glioma xenografts. **f** Weight of the indicated glioma xenograft tumors. n = 5, **, *P* < 0.01. **g** Hematoxylin and eosin staining of tumor tissues samples from indicated groups and detection of GLI2, ARHGEF16, Ki76, and MMP9 protein levels by immunohistochemistry. **h** The decreased ARHGEF16 protein level in U87 GLI2A + sh-ARHGEF16 xenografts compared to U87 GLI2A + sh-Control xenografts

Based on the above observations, we speculated that ARHGEF16 is an effector of GLI2 in the context of glioma progression. To test this hypothesis, we knocked down *ARHGEF16* in GLI2-overexpressing U87 cells. It was then confirmed that the increase in ARHGEF16 was abrogated at the mRNA (Additional file 2: Figure S1D) and protein (Additional file 2: Figure S1E) levels. Moreover, cell migration (Fig. 5a) and proliferation (Fig. 5b, c) were enhanced in GLI2A + sh-Control U87 cells relative to Control+sh-Control U87 cells, suggesting that GLI2 promotes glioma cell migration and proliferation through ARHGEF16. This was supported by the observation that these observed increases were abrogated by *ARHGEF16* knockdown (Fig. 5a-c).

We investigated whether the above findings are applicable in vivo with a mouse tumor xenograft model. Control+sh-Control, GLI2A + sh-Control or GLI2A + sh-ARHGEF16 U87 cells were subcutaneously injected into the flanks of nude mice. Compared to control group, the tumor xenografts of the GLI2A + sh-Control developed much more rapidly, but was significantly retarded by ARHGEF16 knockdown in GLI2A + sh-ARHGEF16 group (Fig. 5d). The tumors of the GLI2A + sh-ARHGEF16 group were also smaller than those of GLI2A + sh-Control group (Fig. 5e, f), and GLI2A + sh-ARHGEF16 xenografts expressed lower protein levels of ARHGEF16 as well as Ki67 (proliferation marker) and MMP9 (marker of cell invasion) (Fig. 5g), as determined by immunohistochemistry. The decreased ARHGEF16 protein level in GLI2A + sh-ARHGEF16 xenograft was also validated via western blotting (Fig. 5h). These results indicate that GLI2/ARHGEF16 signaling promotes glioma progression.

### Blockade of GLI2 suppresses glioma growth

**Fig. 6:**
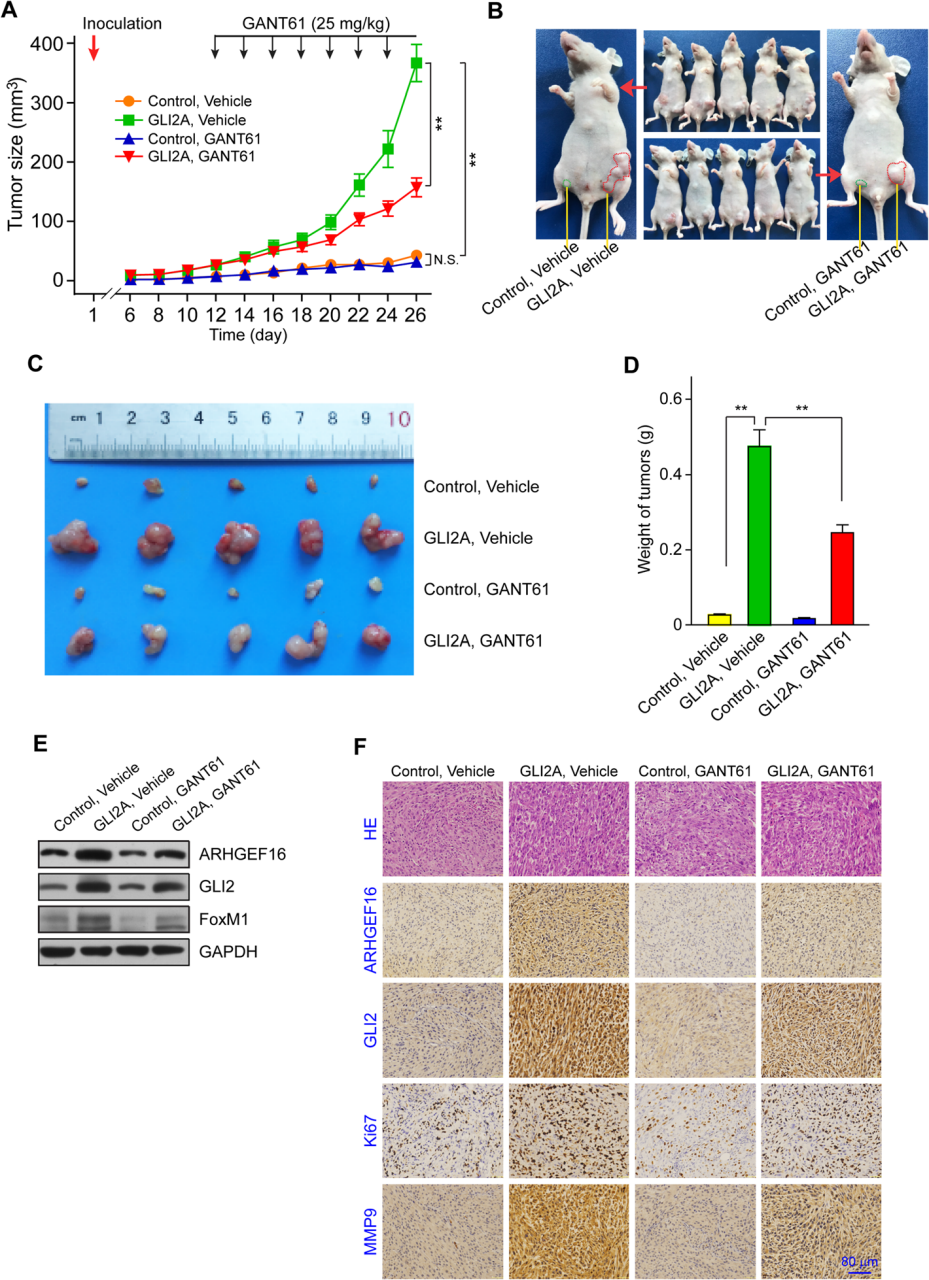
GLI2 inhibition suppresses glioma growth. **a** Growth curves of the indicated groups of U87 glioma xenografts treated with vehicle or GANT61. n = 5, **, *P* < 0.01. **b** Images of tumor-bearing mice. **c** Harvested glioma xenografts formed by indicated U87 glioma cell lines and treatment. **d** Weight of glioma xenograft tumors. n = 5, **, *P* < 0.01. **e** Validation of the decreased ARHGEF16 and GLI2 protein levels resulting from GANT61 treatment in Control, GANT61 and GLI2A, GANT61 groups compared to the vehicle groups, while FoxM1 was used as positive control. **f** Hematoxylin and eosin staining of tumor tissues samples from indicated groups and detection of GLI2, ARHGEF16, Ki76, and matrix MMP9 protein levels by immunohistochemistry

To address the effects of GLI2 inhibition on glioma in a pre-clinical context, GLI2A or Control U87 cells were subcutaneously injected into the flanks of athymic nude mice. Six days after inoculation of tumor cells, the mice were randomly divided into two groups and treated with vehicle only or GANT61, and in the meantime, the tumor size was measured as indicated in Fig. 6a. Tumor xenografts in GLI2A, vehicle group developed much more rapidly than those of the control, vehicle group, while GANT61 greatly suppressed tumor growth due to GLI2 inhibition, as shown by the retarded tumor xenografts (Fig. 6a, b) and the reduced tumor size (Fig. 6c) and weight (Fig. 6d) in GLI2A, GANT61 group compared to those in GLI2A, vehicle group; the decreased protein levels of GLI2 and ARHGEF16 resulting from GANT61 treatment in Control, GANT61 and GLI2A, GANT61 groups compared to the vehicle groups were confirmed via western blotting (Fig. 6e). Furthermore, the effects of GLI2 inhibition on glioma growth were supported by the lower protein levels of ARHGEF16 as well as Ki67 and MMP9 in GLI2A, GANT61 group than in GLI2A, vehicle group, as determined by immunohistochemistry (Fig. 6f). These results indicate that glioma progression can be suppressed by the blockade of GLI2.

### CKAP5 interacts with ARHGEF16 to promote cell migration and proliferation in glioma

**Fig. 7:**
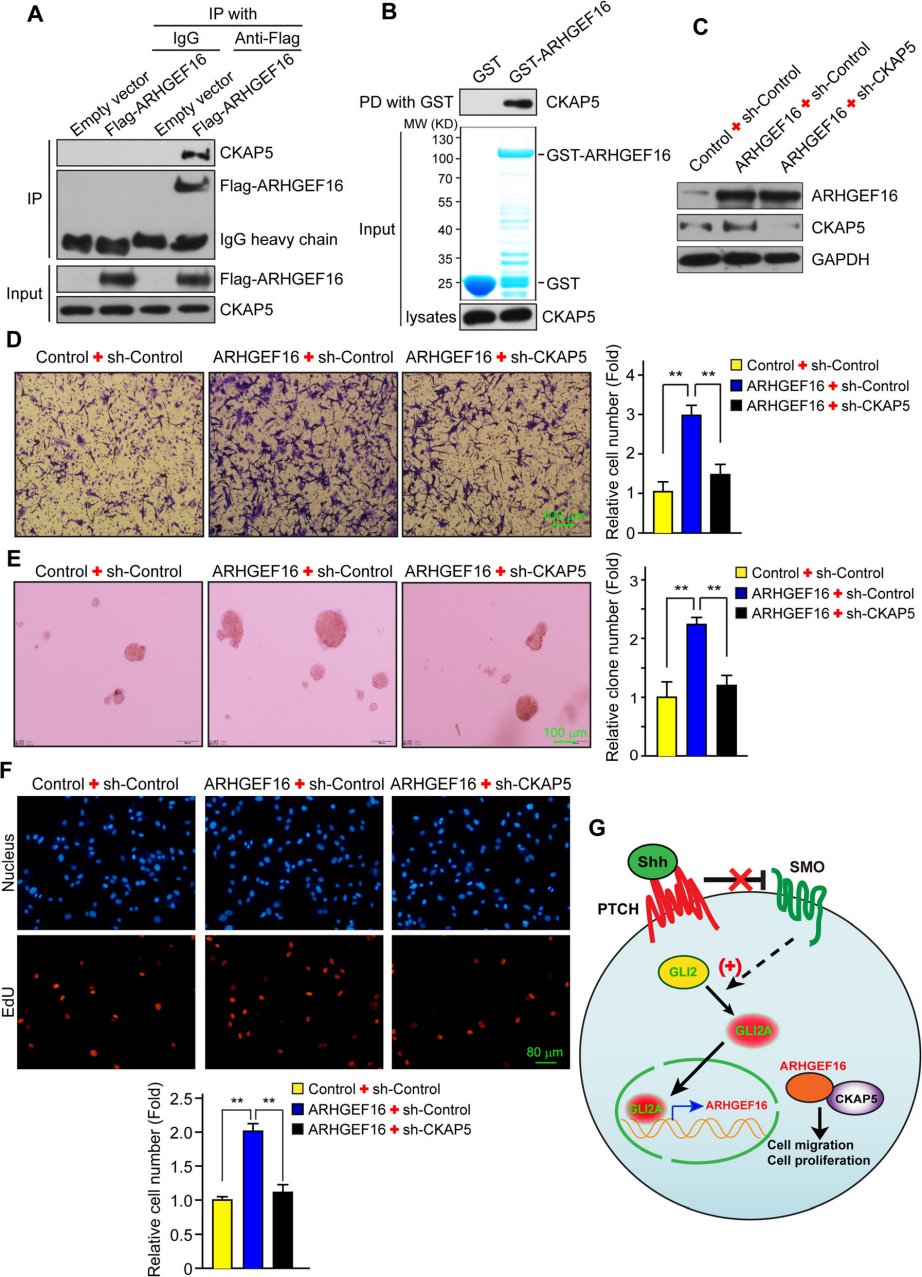
CKAP5 interacts with ARHGEF16 to regulate glioma cell migration and proliferation. **a**, **b** Examination of the interaction between ARHGEF16 and CKAP5 by immunoprecipitation (**a**) and GST pull-down (**b**). **c** Detection of ARHGEF16 and CKAP5 expression levels in Control+sh-Control, AREHGEF16 + sh-Control, and ARHGEF16 + sh-CKAP5 U87 cells by western blotting. **d** Migratory capacity of Control+sh-Control, AREHGEF16 + sh-Control, and ARHGEF16 + sh-CKAP5 U87 cells was determined with the transwell migration. n = 3, **, *P* < 0.01. **e**, **f** Soft agar colony formation assay (**e**) and EdU assay (**f**) to compare the proliferative capacity of the indicated U87 cells and their corresponding control cells. n = 3, **, *P* < 0.01. **g** The working model of this study. GLI2 directly activates ARHGEF16 transcription, which acts in conjunction with CKAP5 to promote glioma cell migration and proliferation induced by GLI2

To clarify the mechanistic basis for the cancer-promoting function of ARHGEF16, a GAL4 yeast two-hybrid screen was carried out using ARHGEF16 as the bait protein. The results of the assay showed that CKAP5 interacts with ARHGEF16, which was confirmed in co-IP (Fig. 7a) and GST pull-down (Fig. 7b) assays in U87 cells.

CKAP5 promotes tumorigenesis by regulating the assembly and stability of the mitotic spindle [40, 41]. To investigate the role of CKAP5 in the glioma-promoting functions of ARHGEF16, we knocked down *CKAP5* while overexpressing ARHGEF16 in U87 glioma cells. ARHGEF16 and CKAP5 protein levels were confirmed by western blotting (Fig. 7c). We then carried out transwell migration (Fig. 7d), soft-agar colony formation (Fig. 7e) and EdU (Fig. 7f) assays to evaluate the migratory and proliferative capacities of these cells. ARHGEF16 + sh-Control U87 cells showed enhanced migration (Fig. 7d) and proliferation (Fig. 7e, f) as compared to Control+sh-Control U87 cells. However, these increases were abrogated by *CKAP5* knockdown. Thus, CKAP5 acts in conjunction with ARHGEF16 to promote glioma cell migration and proliferation induced by GLI2 (Fig. 7g).

## Discussion

Glioblastoma is the most common type of malignant tumor in central nervous system and has a high recurrence rate owing to a strong capacity for migration and proliferation [20, 21]. The Hh signaling pathway regulates tissue patterning during embryogenesis and contributes to the maintenance of adult tissues [1, 2]. Numerous studies have shown that the Hh signaling pathway promotes gliomagenesis by maintaining the cancer stem cell pool [42–44]. The oncogenic effects of Hh signaling are mediated by the target genes of GLI transcription factors [15], suggesting that GLI inhibitors could possibly be used for cancer therapy [18, 45].

In this study we identified *ARHGEF16* as a target gene of GLI2 in glioma cells. We found that GLI2 binds to the *ARHGEF16* promoter to activate gene transcription, suggesting that *ARHGEF16* is a novel target gene of GLI2. Among the three GLI transcription factors in mammals, GLI2 plays the most important role; in mice, GLI2 deficiency results in severe developmental defects and embryonic lethality [46–49]. ARHGEF16 is a GEF of the Rho GTPase family [50] whose members modulate cell morphogenesis, proliferation, invasion, and survival through regulation of the actin cytoskeleton [27, 28]. Accordingly, we found that ARHGEF16 enhanced migration and proliferation in glioma cells. ARHGEF16, which is also known as Ephexin 4, can bind to the cytoplasmic region of Ephrin receptor [50]. Ephrin signaling plays a key role in cellular repulsion, attraction, and migration by controlling local cytoskeletal dynamics through Ephexin proteins and Rho GTPases [51–53]. Our finding that *ARHGEF16* is a target of GLI2 provides the first evidence of potential cross-talk between the Hh and Ephrin signaling pathways in glioma development.

Our study also identified CKAP5 as an ARHGEF16-interacting protein. CKAP5 is an evolutionarily conserved member of the XMAP215 family of microtubule-associated protein [54–56] that is highly expressed in the mammalian brain [57] but has also been found to be upregulated in colonic and hepatic tumors [58]. It is also necessary for the survival of head and neck cancer as well as lung cancer cells [59], and its expression level in liver cancer is an independent prognostic factor for both progression-free and overall survival, with a significant correlation between high CKAP5 level and poor prognosis [41]. CKAP5 is required for the assembly and maintenance of the spindle apparatus during mitosis and meiosis and associated processes such as chromosome segregation and apoptosis [40, 60], and its deletion leads to the formation of multipolar spindles and cell death [61]. The identification of CKAP5 as an ARHGEF16-interacting protein in this study suggests that regulation of spindle integrity is important for glioma cell proliferation and migration. Further studies are needed to explore the mechanistic basis for the interaction between CKAP5 and ARHGEF16.

Given the oncogenic effects of some GEFs, it is in principle possible to target the oncogenic GEFs and CKAP5 for cancer therapy. Though no compound targeting ARHGEF16 or CKAP5 has been reported currently, some compounds targeting GEFs have been developed based on the comprehensive insights into the structural basis of GEFs and small G proteins interaction [62, 63]. For instance, brefeldin A can inhibit the small ARF family members of the small G protein superfamily through stabilizing the ARF-GDP-GEF complex and thus trapping the GEF in an unproductive sate with its substrate [64]; in addition, the compound NSC23766 can block the interaction between Rac and Tiam or Trio, both of which are the GEFs for Rho small G protein family, to inhibit Rac activity, thus presenting anticancer effects [65]. The strategies to inhibit or stabilize the interaction of ARHGEF16 and its G proteins, along with GLI2 inhibition, can be used for cancer therapy.

## Conclusions

In summary, we showed that *ARHGEF16* was a novel target gene of GLI2 and identified CKAP5 as an ARHGEF16-interacting protein. Our results indicate that the GLI2/ARHGEF16/CKAP5 axis promotes glioma progression by enhancing tumor cell migration and proliferation and could therefore serve as a therapeutic target for glioma treatment.

## Additional files

Additional file 1: **Table S1.** Primer sequences for ARHGEF16-Luc reporters. **Table S2.** Target sequences of gene-silencing constructs. **Table S3.** Primer sequences for qPCR. (DOC 47 kb)

Additional file 2: **Figure S1.** (A-C) Efficiency of sh-ARHGEF16 or sh-CKAP5 constructs were examined in 293 T cells via real-time qPCR. The most efficient sequences, sh-ARHGEF16 #3 (Red) and sh-CKAP5 #1 (Blue), were used in this study (see in Additional file 1: Table S2). (D, E) ARHGEF16 mRNA (D) and protein (E) levels in Control+ sh-Control, GLI2A + sh-Control, and GLI2A + sh-ARHGEF16 U87 cells as determined by real time qPCR and Western blotting, respectively *n* = 3, **, *P* < 0.01. (TIF 1196 kb)

## Abbreviations

ARHGEF16: Rho guanine nucleotide exchange factor 16
CKAP5: Cytoskeleton-associated protein 5
GAP: GTPase-activating proteins
GEFs: Guanine nucleotide exchange factors
GLI: Glioma-associated oncogene transcription factors
Hh: Hedgehog
PTCH: Patched
SuFu: Suppressor of fused
TSS: Transcription start site
