# Supplementary material for: GLI2 promotes cell proliferation and migration through transcriptional activation of ARHGEF16 in human glioma cells
Source: J Exp Clin Cancer Res. 2018 Oct 11;37:247. doi: 10.1186/s13046-018-0917-x (PMC6180656; doi:10.1186/s13046-018-0917-x)
Supplement: Supplementary file 1 — Table S1. Primer sequences for ARHGEF16-Luc reporters. Table S2. Target sequences of gene-silencing constructs. Table S3. Primer sequences for qPCR. (DOC 47 kb) [file 13046_2018_917_MOESM1_ESM.doc]

**Table S1 Primer sequences for ARHGEF16-Luc reporters**

| Primer name | Sequence |
| --- | --- |
| Frag-I-Forward | 5’-CTAGCTAGCTGGACACCTACCCTGGAAGGTCGA-3’ |
| Frag-I-Reverse | 5’-CGCTCGAGATGATGGGTACTGCACCGACGAG-3’ |
| Frag-II-Forward | 5’-CTAGCTAGCAGCACTCAGTGGGAATCAGGAACC-3’ |
| Frag-II-Reverse | 5’-CGCTCGAGTCACAGAGAAGGACCGAGTCCTG-3’ |
| Frag-III-Forward | 5’-CCGCTCGAGTAAGTCACCTGGACCAAAGTCAC-3’ |
| Frag-III-Reverse | 5’-CCCAAGCTTCCTGTTATCTGGATCAAGGTCC-3’ |

**Table S2. Target sequences of gene-silencing constructs**

| Name | Sequence |
| --- | --- |
| sh-ARHGEF16 | 5’-AGGACTTTGCCCGCTTCATCA-3’ |
| sh-CKAP5 | 5’-CTAGATGAAGTGGCAGGTATT-3’ |

**Table S**3. Primer sequences for qPCR

| Primer name | Sequence |
| --- | --- |
| ARHGEF16- Forward | 5’-GATACGCTCTGCCTCAAGAC-3’ |
| ARHGEF16-Reverse | 5’-GGGACTTGACCTTGCTGAAG-3’ |
| GLI1-Forward | 5’-GCAGTAAAGCCTTCAGCAATG-3’ |
| GLI1-Reverse | 5’-GTCTTGACATGTTTTCGCAGC-3’ |
| GLI2-Forward | 5’-CTCAAGGAAGATCTGGACAGG-3’ |
| GLI2-Reverse | 5’-GATGTGCTCGTTGTTGATGTG-3’ |
| SOX2-Forward | 5’-TTCACATGTCCCAGCACTAC-3’ |
| SOX2-Reverse | 5’-TCCATGCTGTTTCTTACTCTCC-3’ |
| PTCH1-Forward | 5’-ACTCCCAAGCAAATGTACGAG-3’ |
| PTCH1-Reverse | 5’-TTGAGTGGAGTTCTGTGCG-3’ |
| GAPDH-Forward: | 5’-CAGGGCTGCTTTTAACTCTG-3’ |
| GAPDH-Reverse | 5’-GATTTTGGAGGGATCTCGC-3’ |
