# Supplementary figures and images for: GLI2 promotes cell proliferation and migration through transcriptional activation of ARHGEF16 in human glioma cells
Source: J Exp Clin Cancer Res. 2018 Oct 11;37:247. doi: 10.1186/s13046-018-0917-x (PMC6180656; doi:10.1186/s13046-018-0917-x)

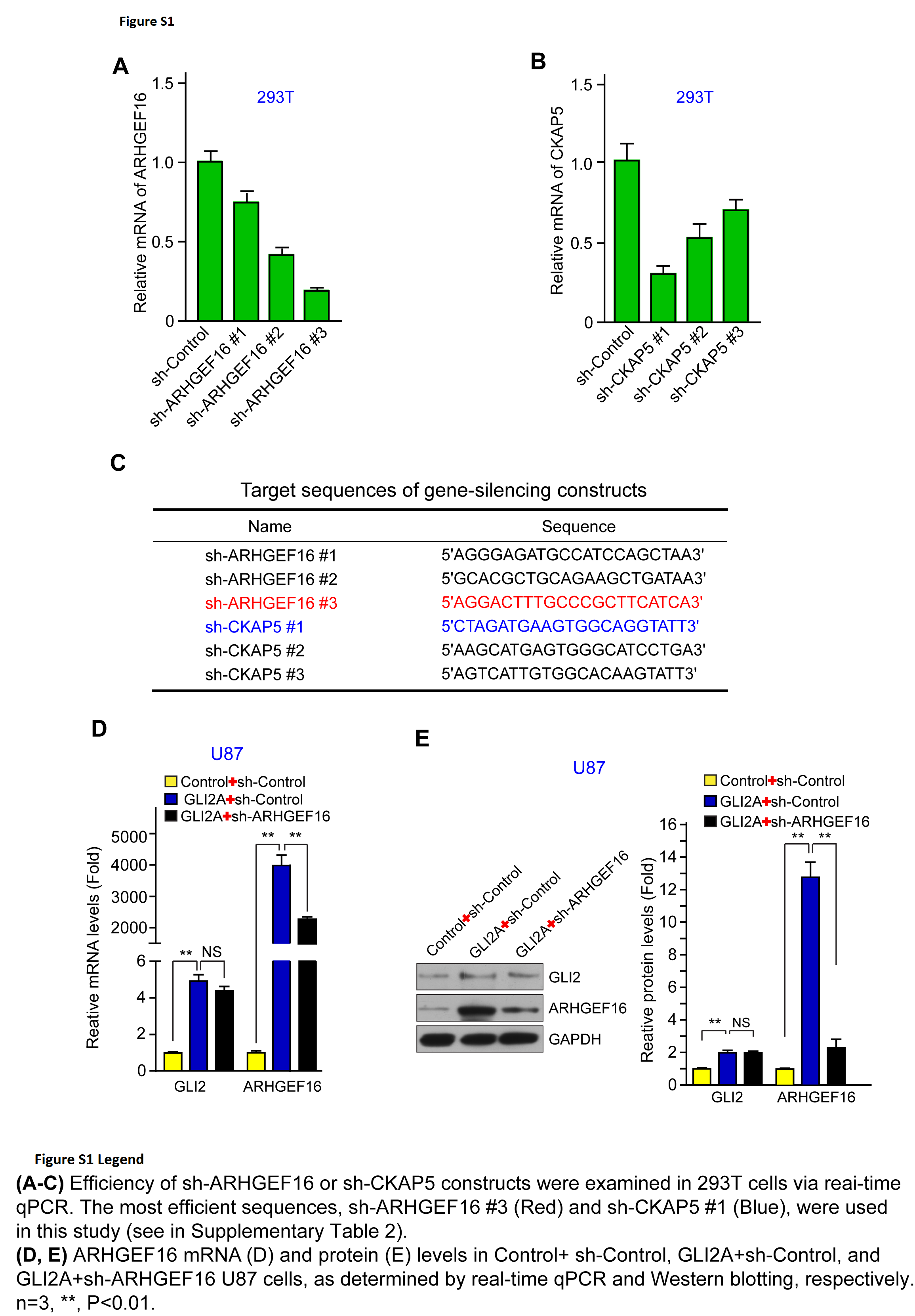

Supplement: Supplementary file 2 — Figure S1. (A-C) Efficiency of sh-ARHGEF16 or sh-CKAP5 constructs were examined in 293 T cells via real-time qPCR. The most efficient sequences, sh-ARHGEF16 #3 (Red) and sh-CKAP5 #1 (Blue), were used in this study (see in Additional file 1: Table S2). (D, E) ARHGEF16 mRNA (D) and protein (E) levels in Control+ sh-Control, GLI2A + sh-Control, and GLI2A + sh-ARHGEF16 U87 cells as determined by real time qPCR and Western blotting, respectively n = 3, **, P < 0.01. (TIF 1196 kb) [file 13046_2018_917_MOESM2_ESM.tif]
